# A robust operational model for predicting where tropical cyclone waves damage coral reefs

**DOI:** 10.1038/srep26009

**Published:** 2016-05-17

**Authors:** Marji Puotinen, Jeffrey A. Maynard, Roger Beeden, Ben Radford, Gareth J. Williams

**Affiliations:** 1 Australian Institute of Marine Science, 35 Stirling Highway, Crawley, Western Australia 6009, Australia; 2 SymbioSeas and the Marine Applied Research Center, Wilmington NC 28411, United States of America; 3 Laboratories d’Excellence ≪CORAIL≫ USR 3278 CNRS – EPHE, CRIOBE, Papetoai, Moorea, Polyne’sie Francaise; 4 Great Barrier Reef Marine Park Authority, Townsville, Australia; 5 School of Ocean Sciences, Bangor University, Menai Bridge, Anglesey LL59 5AB, UK

## Abstract

Tropical cyclone (TC) waves can severely damage coral reefs. Models that predict where to find such damage (the ‘damage zone’) enable reef managers to: 1) target management responses after major TCs in near-real time to promote recovery at severely damaged sites; and 2) identify spatial patterns in historic TC exposure to explain habitat condition trajectories. For damage models to meet these needs, they must be valid for TCs of varying intensity, circulation size and duration. Here, we map damage zones for 46 TCs that crossed Australia’s Great Barrier Reef from 1985–2015 using three models – including one we develop which extends the capability of the others. We ground truth model performance with field data of wave damage from seven TCs of varying characteristics. The model we develop (4MW) out-performed the other models at capturing all incidences of known damage. The next best performing model (AHF) both under-predicted and over-predicted damage for TCs of various types. 4MW and AHF produce strikingly different spatial and temporal patterns of damage potential when used to reconstruct past TCs from 1985–2015. The 4MW model greatly enhances both of the main capabilities TC damage models provide to managers, and is useful wherever TCs and coral reefs co-occur.

Tropical cyclones (hurricanes, typhoons; TCs) generate very rough seas that can severely damage vulnerable marine biota such as coral reefs. Reefs have evolved with intermittent TCs and other natural stressors over millennia^1^, but recovery is now increasingly compromised by chronic exposure to multiple stressors threatening coral reef resilience^2^. Given this, TCs can be a major driver of ecological condition in areas where they regularly occur^3^, including much of the world’s tropical regions.

The severity of physical wave damage to coral reef communities from TCs ranges from broken colony tips and branches to dislodgement and removal of entire colonies to removal of parts of the reef structure itself (e.g., Fig. 1). The type of impact varies based on the combination of the intensity of the waves as well as their duration near a given reef. For example, brief exposure (~an hour) to very powerful (larger) waves can immediately break corals and dislodge colonies, while such waves need to persist longer to remove entire sections of reef framework. These impacts can alter coral community structure through selective mortality of vulnerable growth forms and differential recruitment during recovery^4^. Full recovery from the most severe damage can take decades to centuries^4^, and when combined with other stressors, may lead to a permanent loss of resilience as has been observed in Jamaica^5^ and the Great Barrier Reef (GBR^6^, see Fig. 2).

A single TC can expose hundreds of reefs to damaging waves in locations that are typically located far from human population centres. This makes field assessments of damage and recovery from large and/or severe cyclones more expensive than when TC impacts are concentrated within a smaller area closer to a port. The entire area potentially damaged by a given TC can rarely be surveyed, creating difficult trade-offs when spatially allocating field work effort. Therefore, spatial models that approximate the TC high energy zone are used to predict the location of severe damage from a given TC, which we term the ‘predicted damage zone’. Such models are vital for reef (or other natural resource) managers for two key reasons. We alluded to the first already - predicted damage zones can be constructed in near-real time after a TC occurs. These zones can inform impact assessments and therefore identify targets for management actions that promote recovery (e.g. temporary closures, improving water quality or even active restoration and rehabilitation). Secondly, damage zone models can identify spatial patterns in historic TC exposure that help explain habitat condition trajectories, as has been done for the Caribbean^7^ and the GBR^3^.

The spatial distribution of wave damage from cyclones is always highly patchy^8^. This is true even for very intense TCs, as has been shown in Jamaica^9^ and the GBR^10^. This occurs because myriad local scale^11^ and regional scale^12^ factors affect the vulnerability of corals to damage. Even within a TC’s highest energy zone near the TC track, some invulnerable corals may remain undamaged. For this reason, any cyclone reef damage model will always include a high rate of false positives (where damage is predicted but none actually occurs), no matter how conservatively thresholds are set. This makes attempts to ‘tune’ thresholds for damage using field data of actual damage problematic because thresholds defined in such a way tend to be too specific to the characteristics of a given cyclone as well as very sensitive to the spatial distribution of the field survey sites with respect to the cyclone track (and the side of the track). For all of these reasons, it is easy to ‘overfit’ a model to the local context of the sites that happened to be surveyed. Models fitted in such a way are not robust to use with other cyclones that have differing intensity, size, translation speed, or duration near reefs, and so cannot be used by managers as operational tools. Consequently, cyclone damage models focus on defining the spatial zone where TC conditions were sufficiently intense to damage vulnerable corals, accepting that some corals within this zone will not actually be damaged. A reasonable goal for predicted damage zones is to correctly identify as much observed severe damage as possible (high true positive rate) while maintaining a reasonable level of overall model accuracy. To achieve this, we suggest a minimum true positive rate (also termed ‘recall’, ‘sensitivity’^13^) of 0.9, and an AUC (area under curve) of at least 0.7 for ROC (receiver operating characteristic) accuracy (balance between true positive and true negative rates).

The simplest models for predicting TC damage assume that severe impacts occur within a single threshold distance of a TC track. Several authors have defined such thresholds, including 65 km^14^ in the Caribbean and 35 km^15^,^16^ in the GBR. Defining a zone this way can both underestimate and overestimate the spatial extent of actual damage because TC wave heights are asymmetrical around the track^17^. To account for this, some models vary the threshold distance by side of the track, such as^15^ and^18^ in the GBR and^19^ in the Caribbean. Given that more intense TCs can generate higher waves, some authors (such as^7^ and^19^ in the Caribbean) use longer threshold distances for them than weak TCs. This approach assumes that more intense TCs are larger than weaker TCs, which is not always the case, especially in the East Pacific and Southern Hemisphere^20^. Finally, the intensity, size and translation speed of a given TC continually vary along its track and interact to control both the magnitude and extent of extreme conditions. This means that a threshold distance within which reef damage could be expected to occur will also vary continually along the track, and the only way to define accurate thresholds is to use the equations found in a parametric cyclone wind model. An alternative approach uses such models^21^ to reconstruct the spatial distribution of TC wind speeds around the track for every hour of the storm, and then field data of wave damage to establish thresholds in maximum wind speed and duration to define a damage zone^22^,^23^. The approach we apply builds on this to adjust for the fact that wind-generated waves - not the winds themselves - cause the damage to reefs. The overall sea state (distribution of waves of various heights) created by any application of wind on water over time depends both on the duration of winds of various speeds and on fetch - how much open water exists given the direction of incoming wind^24^. Our proposed damage zone model builds on early work in Jamaica^25^ to predict the spatial distribution of a sea state rough enough to severely damage reefs during a TC. The same model could be applied to any wave-vulnerable biota of interest by specifying an appropriate threshold sea state for that biota.

Here, we map damage zones from each of 46 TCs that crossed Australia’s GBR from 1985 to 2015 using three models. One is based on distance and intensity thresholds (AHF), and the second is based on wind speed and duration (FAB). The third - our proposed model (4MW) - predicts where sea states rough enough to damage reefs were possible. We calculate true positive rates (sensitivity) and measure partial AUC (overall accuracy) for severe damage versus none for seven TCs for which we have extensive field data of TC wave damage. We also compare the spatial extent of the damage zones produced by each model and the percentage of GBR reef area that falls within these zones, for each of the 46 TCs. We then assess how the spatial and temporal trends generated by combining these data over the 30-year time series differ between the top two performing models. Finally, we use the results to assess the consequences of model choice by scientists and managers when considering the recent (just after a TC) and past implications of TC damage on reefs.

## Methods

### Assessing model performance in predicting severe damage

**Figure 1:**
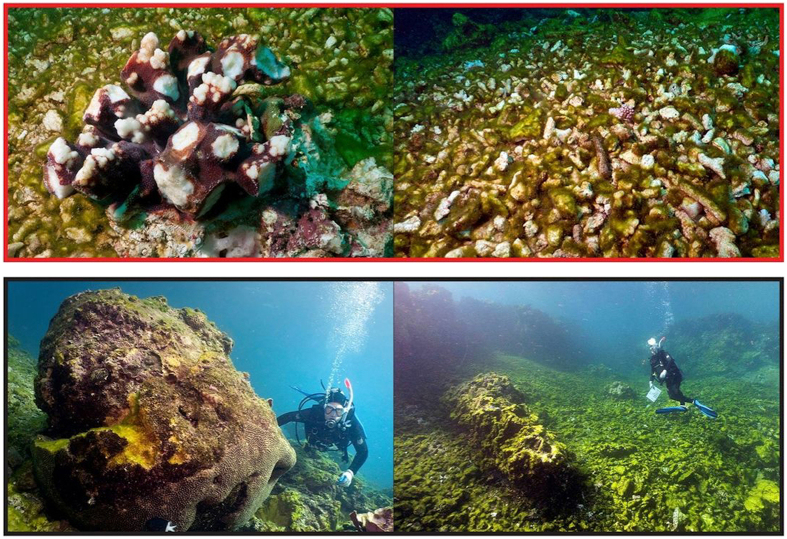
Photos representative of severe damage for our analyses of model performance in predicting where cyclones damage coral reefs. These were taken by Roger Beeden following TC Yasi, which crossed the GBR February 3, 2011 (see^10^, Fig. 4). The upper panel shows ‘severe’ damage where many (31–50%) colonies are dead or removed, there is extensive scarring by debris, there are rubble fields littered with small live coral fragments, soft corals are severely damaged or removed, and some large coral colonies are dislodged. The lower panel shows ‘extreme’ damage where most (51–100%) corals are broken or removed, soft corals are removed, and many large coral colonies are dislodged.

As corals are continually subject to mortality from routine processes^26^, confidently attributing observed damage to TC waves becomes increasingly difficult as damage becomes less severe. Therefore, we focused on predicting severe damage (Fig. 1), after which either many coral colonies are dead or removed and some large colonies are dislodged (severe), or most corals are broken, dead or removed and many large colonies are dislodged (extreme; examples in^10^).

**Figure 2:**
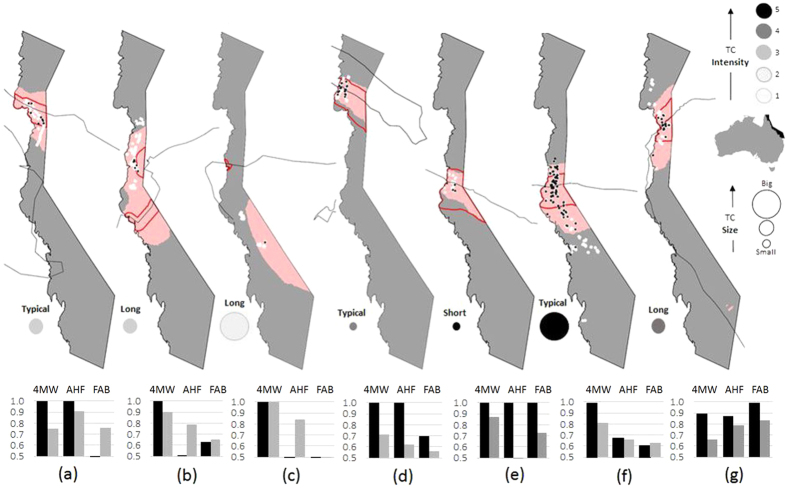
The performance of three models (4MW, AHF, FAB – see Table 1) in predicting severe coral damage in the GBR for 7 TCs for which field data were available: (a) Ivor – 1990; (b) Joy – 1990, (c) Justin – 1997, (d) Ingrid – 2005, (e) Larry – 2006, (f) Yasi – 2011, and (g) Ita–2014. The TC tracked along the thin black line. The 4MW and AHF predicted damage zones are shaded pink and outlined in red, respectively. Field survey sites are shown by black circles (severe damage) and white squares (no severe damage). The true positive rate (how well damage is detected) and overall model accuracy (pAUC) are shown for each model below each map. ArcGIS 10.2 software (https://www.arcgis.com/features/) was used to create the maps.

North-east Australia’s Great Barrier Reef (GBR, Fig. 2) provides an ideal case study for testing the performance of damage zone models as applied to corals. Broad-scale surveys have been conducted to assess the severity, extent and type of TC wave damage following 7 TCs with varying intensities, sizes and durations between 1990 and 2014, as detailed in Fig. 2. This is in contrast to the rest of the world, where most such surveys have focused on one or only a few reefs (e.g.^27^ Guam^11^, Jamaica^28^, French Polynesia^29^, US Virgin Islands^30^, Hawaii^31^, Mexico^32^, Netherlands Antilles^33^, Florida Keys), or used coral cover loss measured as an indicator of TC damage without measuring such damage directly^7^. The maps in Fig. 2 show where sites were surveyed for each TC (black dots – severe damage, white dots – no damage or damage that was not severe). The GBR surveys include: Ivor (1990^15^, n = 46 sites on^35^ reefs), Joy (1990 – Ayling unpublished data, n = 199 sites on^46^ reefs), Justin (1997 – Puotinen unpublished data, n = 54 sites on 15 reefs), Ingrid (2005^23^, n = 490 sites on 32 reefs), Larry (2006 – Fabricius unpublished data, n = 305 sites on 23 reefs), Yasi (2011^10^, n = 841 surveys on 70 reefs), and Ita (2014 – GBR Marine Park Authority unpublished data, n = 315 surveys on 31 reefs). Each survey used manta tows to record how severe and widespread damage was along a series of 2 minute transects. For cyclones Ivor, Joy, Justin, Ingrid and Larry, damage severity was recorded for up to each of eight different types of damage (dislodgment of massive colonies, breakage, sand burial, debris scarring, exfoliation, stripping of soft corals and trenching). This was done based on the percentage of colonies that were damaged for each damage type, ranging from a value of 0 (none) to 5 (90–100% of colonies damaged). For these cyclones, we classified each site as severely damaged if it scored a damage severity value of at least 3 (40–60% of colonies damaged) for at least 3 of the 8 possible damage types. For cyclones Yasi and Ita, damage severity was recorded in 3 levels based on whether damage was constrained to colony tips, entire colonies or entire sections of reef. The prevalence of these levels of damage was used to estimate how widespread each type of damage was, and then five categories of overall damage severity were defined (see^10^). For these TCs, we defined severe damage as that falling into the ‘severe damage’ (31–50% colonies dead or removed, extensive scarring by debris, rubble fields littered with small live coral fragments, soft corals severely damaged or removed, some large coral colonies dislodged) and ‘extreme damage’ categories (51–100% corals broken or removed, soft corals removed and many large coral colonies dislodged). See Fig. 1 for pictures of these damage levels on reefs.

We tested the performance of each of three damage zone models – two described in the literature (AHF, FAB) and one we developed ourselves (4MW). TC damage models focus on identifying a spatial zone beyond which severe damage should not occur based on exposure to extreme winds and waves capable of damaging vulnerable reefs. Two of the models use severe damage threshold(s) defined *a priori* (4MW, AHF) and one tunes thresholds to observed patterns of damage from field data (FAB). The model we developed (4MW), defines an *a priori* threshold (exposure to a sea state capable of damaging most vulnerable reefs for at least one hour). We call the model 4MW because we define the threshold sea state as where the highest one-third of wave heights in a region over a sustained period of high winds are 4 m or greater, with a maximum wave height of ~10 m (significant wave height = 4 m). Such seas are at least one-third more energetic than calm conditions and have been shown to move entire reef blocks onto the reef flat^34^. We describe development of the 4MW model in detail within the Supplementary Material.

Similarly, we use *a priori* distance and intensity thresholds for severe damage proposed in^19^ to define what we call an Approximate area of Hurricane Force (AHF) winds. The threshold distance from the track that defines the AHF damage zone varies with cyclone intensity (measured as Saffir-Simpson intensity categories, 0 to 5) and the side of the TC track. For example, hurricane force winds are assumed to extend 23.6 km from the left (weak) side of the track and 47.2 km from the right (strong) side of the track for a category 1 hurricane in the northern hemisphere. We construct our version of the AFH damage zone by applying the appropriate threshold distance every hour along the TC track based on the TC intensity, as obtained from the Australian Bureau of Meteorology’s cyclone database (http://www.bom.gov.au). We create a preliminary damage zone for each side of the cyclone track, and then combine the two zones.

Finally, FAB is named for Fabricius *et al*. who used field data of wave damage to reefs from cyclone Ingrid (2005) to define tuned wind speed and duration thresholds for wave damage to inner and middle shelf versus outer shelf reefs on the GBR. We applied these thresholds to the reconstructed hourly wind speed data we generated for each cyclone using a parametric cyclone wind model^21^ to create a FAB damage zone (see Table 1).

We created damage zones for each of the seven TCs for which we have extensive field data of wave damage using each of the three models. Figure 2 shows the 4MW damage zone for each cyclone shaded in pink and that for AHF outlined in red. We then assessed model performance based on two indicators- 1) how well the model detected all known severe damage, and 2) whether it generated an acceptable rate of false positives within the damage zone. For the former, we measured the proportion of real incidences of severe damage that are correctly predicted to be severely damaged (true positive rate, sensitivity). For the latter, we plotted the true positive rate against the true negative rate to create receiver operating characteristic (ROC) curves. ROC curves graphically depict model performance. Models that perform well detect a high rate of true positives as well as true negatives, and when plotted, generate ROC curves located above a diagonal line originating at the origin (0, 0). The area under the ROC curve (AUC) provides a measure of overall accuracy^13^. Because the 7 field data sets varied considerably in sample size (from n = 46 to n = 886), we bootstrapped the data with replacement (10,000 iterations per cyclone per model) to generate an unbiased sample for each cyclone for presence and absence of severe damage. Then we ran a series of partial ROC curves to identify the optimal response threshold across all the models and used it to calculate pAUC (partial area under curve) scaled between 0 and 1 using the R v3.2.4 (R Development Core Team, www.r-project.org) library pROC v0.1.2 for each cyclone for each model. pAUC values greater than or equal to 0.7 indicate a model that adequately balances between true positive and true negative rates to achieve an acceptable overall accuracy. Model skill was deemed acceptable for management purposes if it did well at detecting known severe damage (e.g. met or exceeded a threshold of 0.9 for true positive rate) without compromising overall model quality due to a high false positive rate (e.g. a value of at least 0.7 for pAUC). We identified a ‘top performing’ model for each TC as the model with the highest acceptable (> = 0.9) true positive rate that still had an acceptable cost of false positives (pAUC > = 0.7). Our choice to favor the true positive rate over the true negative rate is based on extensive discussions with coral reef managers about the greater cost (for opportunities and reputation) of a false negative than a false positive [reviewed in^35^,^36^]. Finally, we built TC damage zones for the remaining 39 TCs that tracked near the GBR from 1985 to 2015 and tested whether the percent area of reef in the predicted damage zone differed significantly between models.

### Variability in return times of TC exposure

For the two top performing models, we mapped GBR-wide patterns in the frequency of potential cyclone damage over the 30 year time series (1985–2015). Poisson probabilities for a given pixel being located in a damage zone in a given year were calculated for each 4km pixel in the GBR using the formula:


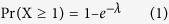


where λ is the annual average number of times a pixel was located in a damage zone from 1985–2015. This follows prior studies that calculated probabilities of TC landfalls^37^,^38^. The annual probabilities were then converted to return times (in years) using the formula:


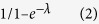


For example, annual probabilities of 100%, 25%, 5%, and 1% equate to return times of 1, 4, 20 and 100 years, respectively. Return times were also calculated for TCs of different characteristics to track anywhere near the GBR over the study period. Coral reef spatial data was sourced from the managing agency of the GBR – the Great Barrier Reef Marine Park Authority (GBRMPA), and was used to examine how: 1) the spatial distribution of return times across the GBR and 2) the total percent area of reef that falls in each of 5 classes of return times, differs between the models.

We tested whether the percentage of total coral reef area across the GBR located inside at least one predicted damage zone per year increased or decreased significantly over the period 1985–2015, and whether this differed between the three models. We tested for trend over time using linear regression with time as a continuous predictor; data were square root transformed to satisfy assumptions of normality. To test for autocorrelation in the time-series data (and thus a lack of data independence) we: 1) examined the model residuals using autocorrelation (ACF) plots, and 2) formally tested for a linear trend in the lag-one correlations of the residuals (e.g. each residual against the subsequent residual) using a linear model (*lm* function in R). In all cases, we found no evidence for temporal autocorrelation in the data.

## Results

### Model performance in predicting severe damage

**Figure 3:**
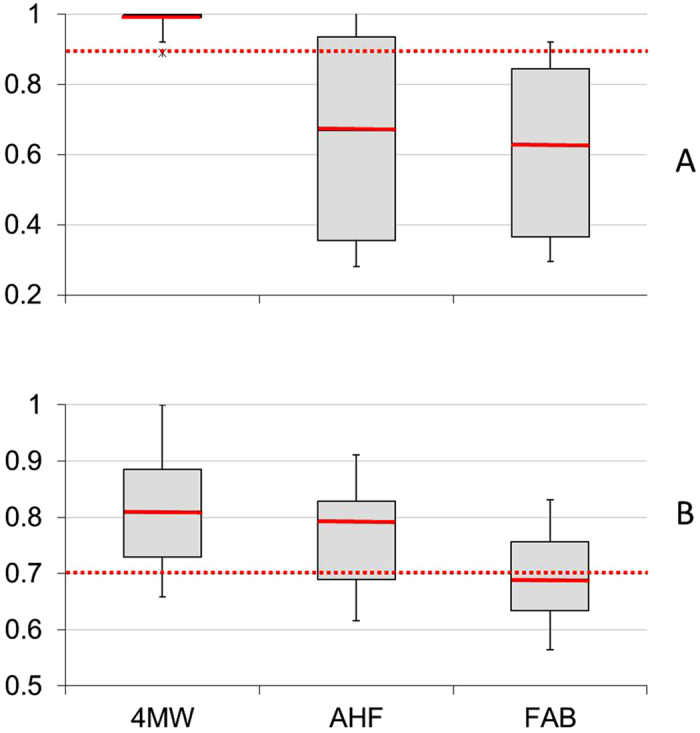
Boxplots of relative performance of three models (4MW, AHF, FAB) for predicting severe cyclone damage for 7 TCs where extensive field survey data was available (see text for description of data sets). (**A**) shows the true positive rate with a performance benchmark of 0.9 (red dotted line). (**B**) shows partial AUC with a standard performance benchmark of 0.7 (red dotted line).

The 4MW model had the highest overall true positive rate and a consistently acceptable pAUC (Fig. 3), outperforming (Fig. 2b,c,f) or matching (Fig. 2a,d,e) the closest other model(s) for all TCs except Ita (Fig. 2g) which was an anomaly due to the local context of some of the sites surveyed (see Supplementary Material section 2). 4MW outperformed the second-best-performing model (AHF) for the large TCs (Justin and Yasi, Fig. 2c,f), and for the unusually long-lived TCs (Joy and Justin, Fig. 2c,b). Although the damage zones generated by 4MW for these TCs were consistently larger than those of AHF or FAB, acceptable pAUC scores (well above the 0.7 benchmark – Fig. 3) suggest that they were not larger at the cost of an unacceptable rise in the false positive rate.

**Figure 4:**
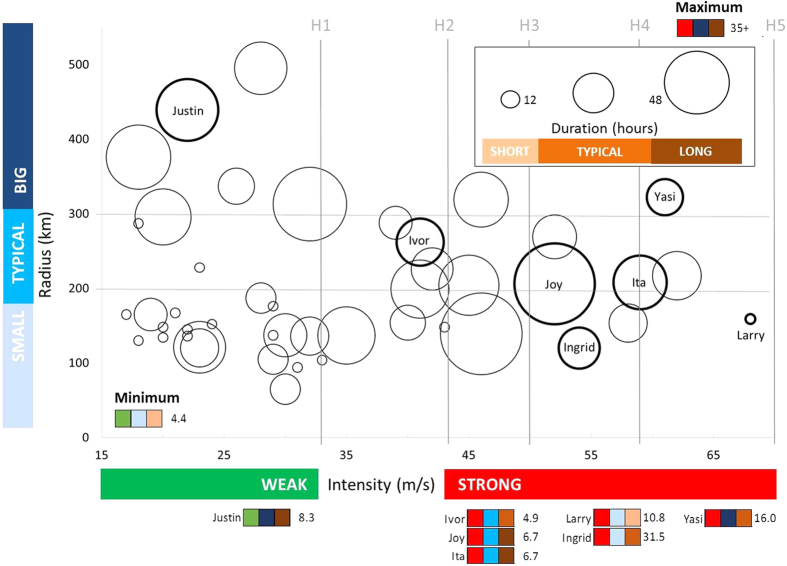
Relative intensity, circulation size, and duration of TCs near the GBR, 1985–2015. Grey lines show TC intensity rankings on the Saffir-Simpson scale. Intensity is shown on the y-axis as maximum wind speed (m s^−1^), and size on the x axis by the mean radius to gale force winds from the TC eye (km). The size of each circle shows the duration of gale force or higher winds (hours) within the GBR. The intensity, size and duration data for each TC in the diagram can be seen in Table 2.

Differences in the ability of the models to accurately predict damage are best exemplified by TC Justin (1997). Justin was the second-largest recorded TC in the GBR over the study period (Fig. 4) while it was located well out in the Coral Sea (Fig. 2c). Justin’s gale force or higher winds (e.g. >17 m s^−1^) persisted for weeks across two-thirds of the GBR even though it was weak (Fig. 4, Table 2) and located 100s of km away from the nearest reefs. Consequently, the model based on distance to the track (AHF) missed all of the observed severe damage (Table 2). Similarly, the model using thresholds based on wind speed and duration (FAB) also missed all of the damage because Justin’s maximum wind speeds were below the thresholds based on normal sized TCs (Table 2). AHF and FAB produced smaller predicted damage zones than 4MW for all TCs that, like Justin, were weak, big and long-lived (Table 2, except Oswald which tracked over land so 4MW predictions were constrained by lack of fetch). Weak, large and long-lived TCs revisit the GBR every 8.3 years (Fig. 4). Detailed comparisons of true positive rates between the models for six other types of TCs (combinations of intensity, circulation size and duration) are provided in the Supplementary Material. The 4MW model is the only model that achieved a true positive rate > = 0.9 for all types of TCs examined (Fig. 3, Table S2). It was the top performer or tied for the top performer based on benchmarks in the true positive rate and pAUC for all cyclones except Ita (Fig. 3, Table S2).

The 4MW model’s damage zones are consistently more spatially extensive than those of the other models (Table 2). The average percent area of reef inside 4MW zones for all 46 TCs was nearly twice that of AHF (7.4% versus 4.3%) and more than three times that of FAB (7.4% versus 1.8%). The percentage of reef area located inside the predicted damage zone varied significantly among models when examined for all TCs from 1985 to 2015 in a permutational ANOVA (Pseudo-F_3,175_ = 13.92, p < 0.0001). All pair-wise comparisons between models were significantly different (p < 0.04).

### Spatial and temporal variability in return times of exposure

**Figure 5:**
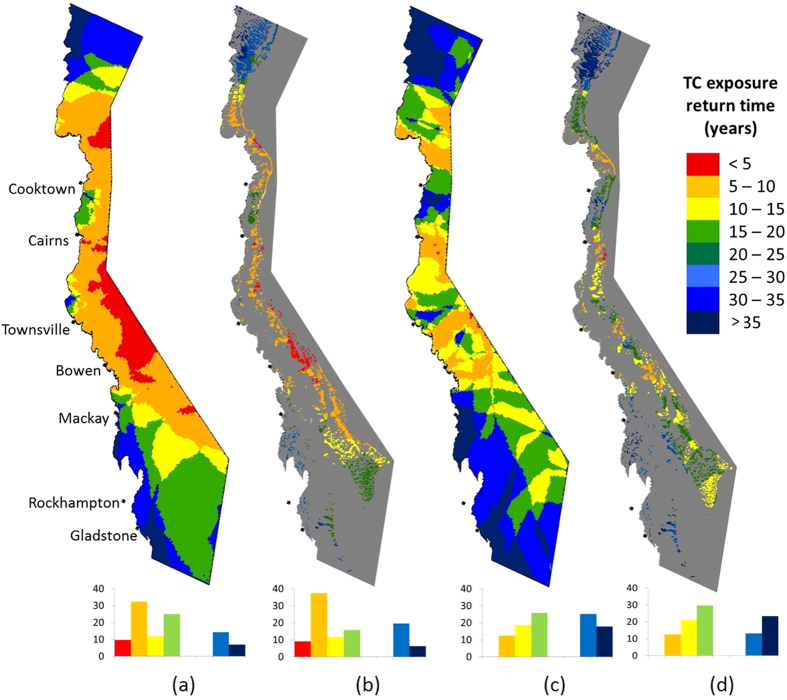
Comparison of spatial patterns of return times for severe TC damage in the GBR, 1985–2015, based on 4MW (**a,b**) and AHF. ((**c,d**) - images on the right are reefs only). Return times indicate the predicted number of years between each time a pixel is located in a predicted damage zone. Pixels with return times greater than 35 years were not located in a TC damage zone from 1985–2015. Methods for 4MW and AHF are in Table 1 and the Supplementary Material. ArcGIS 10.2 software (https://www.arcgis.com/features/) was used to create the maps.

The spatial distribution of exposure to potentially damaging TC activity from 1985 to 2015 was strikingly different when hindcast based on the 4MW (Fig. 5a,b) versus the AHF model (e.g. the model with the next-highest true positive rate, Fig. 5c,d). The most frequent exposure (red areas – return times less than 5 years) was predicted to be much more prevalent across the GBR by 4MW (9% of total reef area) than by AHF (0.08% of total reef area). This makes sense given that AHF under-predicted the area of exposure for the TCs that are likely to cause the most spatially extensive damage – those that are strong and either large (Yasi, Fig. 2f; Table 2) or long-lived (e.g. Joy, Fig. 2b; Table 2) or those that are large *and* long-lived (Justin, Fig. 2c; Table 2). Similarly, AHF predicted a much greater area of the GBR to have never been exposed to damaging TC activity over the study period – 23.5 (Fig. 5d) vs 6.3% (Fig. 5b) of total reef area. The 4MW model predicted a clear concentration of the most frequent exposure (<5 years – red) in the central GBR between Cairns and just south of Bowen on middle and outer shelf reefs (Fig. 5a,b). In contrast, no clear cross-shelf or latitudinal gradients in return times were evident when using the AHF model (Fig. 5c,d). This makes sense given AHF’s focus on TC intensity but not size or duration.

**Figure 6:**
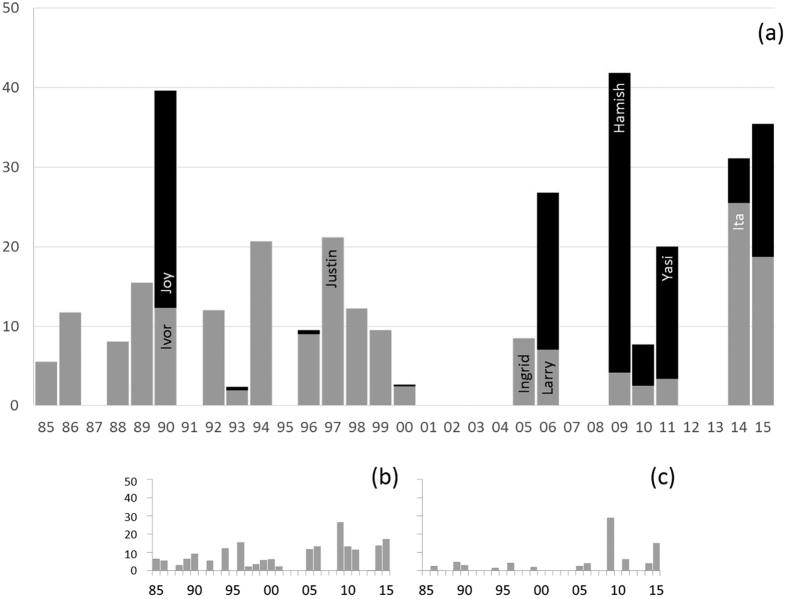
Percentage of the total GBR reef area within severe TC damage zones each year, 1985–2015, for models: (a) 4MW, (b) AHF and (c) FAB. For (**a**), when more than one TC occurred per year, the relative proportion of exposure attributable to each is shown by grey vs black shading. The 7 case study TCs are labelled. Model descriptions are in Table 1 and the Supplementary Material.

The annual percentage of total reef area located inside at least one damage zone from 1985–2015 differed notably across the three models (Fig. 6) – percentages were consistently highest for 4MW (Fig. 6a). The percentage of reef potentially damaged was highly variable over time, particularly for 4MW and AHF (Fig. 6a,b). For 4MW, two clear peaks of activity – the early 1990s and 2006–2015 - are evident. The maximum percent of reef area in a damage zone for a single TC was close to 40% during Hamish (2009) – but usually the highest percentages for a given season came from the combination of two TCs that covered non-overlapping areas, such as intense TCs Marcia (far southern GBR) and Nathan (far northern GBR) in 2015. Because of this bi-modal distribution, we find no significant trend in percent reef area inside a damage zone over time (p = 0.0802, 95% confidence intervals overlap zero). Similarly, no significant trend over time was found for AHF (p = 0.127) or FAB (p = 0.342) and both sets of 95% confidence intervals overlapped zero.

## Discussion

Our results demonstrate that our model that uses reconstructed TC wind speeds, durations and fetch to estimate an *a priori* ‘damaging’ sea state (4MW) outperforms models based on tuned thresholds in wind speed and duration (FAB), and a combination of *a priori* thresholds in distance to the track and intensity (AHF). 4MW was the top performer (or tied for top performer) for all but one of the six types of TCs for which we had field damage data. The disparity between 4MW’s true positive rate and that of the next-best performing model (AHF) was greatest for TCs that were big and/or long-lived. This is critically important as these are the TCs most likely to cause the most spatially extensive wave damage to reefs, and they occur in the GBR regularly (weak, big, long-lived – every 8.3 years; strong, big – every 16 years; strong and long-lived – every 6.7 years). AHF also produced predicted damage zones that were too small for big and long-lived TCs, and likely too large for weak and short-lived TCs. The latter are very common in the GBR (returning every ~3 years). Further, a much greater percentage of GBR reef area was predicted to have been damaged annually for 4MW than the other models. One might easily conclude that severe damage from TCs was virtually non-existent in the GBR prior to 2005 if relying on FAB. However, field damage data from TCs Ivor, Joy and Justin make it clear that this was not at all the case. This difference was driven by the failure of FAB, and to a lesser degree, AHF, to capture damage from TCs that were less intense but longer lasting and/or bigger. We expect the disparity between 4MW and AHF to be even more pronounced in other regions where big TCs are even larger and more frequent than in the GBR, such as the Caribbean and western Pacific^20^. The latter includes the northern part of the Coral Triangle where reefs are particularly diverse^39^ and threatened by a range of anthropogenic local and broad-scale stressors [reviewed in^40^]. Using the 4MW model to understand historic exposure to TC impacts in this area is important future work.

Model choice clearly matters when predicting where to find severe wave damage on reefs. Earlier, we suggested a 0.9 true positive rate and a 0.7 pAUC as benchmarks for the management utility of the TC damage models. We show that 4MW is the only model to achieve the true positive rate benchmark for all seven TCs for which we have field data, representing six types of TCs. For all but TC Ita, pAUC scores above the benchmark confirm that 4MW achieves this at an acceptable cost of false positives. In contrast, FAB meets the true positive rate threshold for only one cyclone (Ita) and so has limited to no management utility. AHF meets the true positive rate with an acceptable rate of false positives only for cyclones that are small or typical in size and duration – missing other cyclone types which occur regularly in the GBR (every 6.7 to 16 years). 4MW is the best model for both of the ways managers can use model results: near-real time informing of research and monitoring, and reconstructing historic exposure to understand trajectories in habitat condition. Running the 4MW model has recently become the best-effort operational tool used by the GBR Marine Park Authority (GBRMPA) to predict where to find severe damage following TCs as an integral part of their Tropical Cyclone Response Plan^41^. Essentially, the 4MW model provides the same enhanced capability to assess and respond to TC impacts on coral reefs as the 5-km Hotspot and Degree Heating Week programs of NOAA Coral Reef Watch^42^ provide for responding to coral bleaching. Like the NOAA products, 4MW predictions represent the *potential* for damage, recognising that actual damage will invariably be patchily distributed due to spatial variability in coral reef susceptibility. Further, 4MW can be applied to other wave-vulnerable biota by determining an appropriate threshold sea state at which severe damage becomes likely for such biota.

The 4MW model also greatly improves our ability to reconstruct historic exposure to TC impacts, enabling studies on the role of TCs in driving ecosystem condition trajectories in the context of other stressors [as exemplified in 3]. We show that mapping spatial patterns in damage return times across the GBR using 4MW versus AHF over the 30-year study period yields strikingly different results. The 4MW model, but not AHF, produces spatial patterns of historic exposure that correspond with what has been found in other regional cyclone studies over different time periods. For example, 4MW yields a similar hotspot of TC activity in the offshore central GBR to that found in several other studies over the recent past^16^,^22^,^43^,^44^,^45^. These differences could be very important when attempting to spatially target management interventions or consider stressor dynamics in marine reserve planning as per^46^ and^47^, or assessing the success of past management actions like the rezoning the GBR Marine Park^48^. One rationale for spatially targeting management interventions^49^, for example, requires identifying reefs that are ‘strong’ (frequently damaged) versus ‘weak’ (infrequently damaged). Using the TC return time data in Fig. 5, reefs located offshore from Bowen would be rated as ‘weak’ by 4MW (TC damage less than every 5 years) but would be rated as ‘strong’ by AHF (TC damage every 15–35 years).

The frequency of TC activity at any given location varies on century or longer time scales^50^, making it difficult to use the dataset presented here (30 years) to assess temporal trends in recent activity. The most extensive temporal dataset of TC activity available for the GBR (5000 years^50^) showed that very intense TCs periodically affected specific locations within the GBR spread between 13–24**°**S (all but the northernmost one-fifth of the GBR) once every 200–300 years. In that context, the recent spate of multiple very intense TCs affecting the GBR within only a decade (Larry 2006, Hamish 2009, Yasi 2011, Ita 2014, Marcia 2015 – Table 2) raises the question of whether the relative proportion of TCs that are intense within the GBR has already increased with global climate change, as suggested by^51^. We found no significant upward trend in the percent reef area exposed to damaging seas (Fig. 5) from 1985–2015 to support this claim. A more robust method would calculate return times and error bounds of potential damage from TCs by mapping damage zones from hundreds of probable ‘synthetic’ TC tracks predicted by global climate models for both current and future climates [as per^52^]. Such data generated on a global basis would show managers which reefs are most likely to be frequently impacted by TCs now and in future climates, as has been done for thermal stress^53^,^54^,^55^ and coral disease^56^. Such robust spatial mapping of broad-scale risk factors is essential input data for reef conservation spatial decision-support frameworks [as per^57^], such as those recently developed for the Coral Triangle^40^.

To be useful to researchers and managers, TC damage models need to be computationally efficient enough to run in near real time after major events, while also sufficiently capturing the spatial extent of severe damage. 4MW falls midway along a continuum of model complexity, from the simplest and fastest distance-based models, to the time consuming, data intensive, fully resolved numerical wind models that drive numerical shallow water wave models (e.g. SWAN^58^). Future work could explore the feasibility of adapting the parametric TC wave model recently developed by^17^ for use in defining a TC damage zone. This has the potential to reduce some of the false positives in the damage zone by more accurately modelling the TC wave field without having to run a numerical wave model. For reefs specifically, false positives could also be reduced by incorporating models of reef structural vulnerability to waves based on factors like coral colony shape^11^. However, the vast size of the GBR severely limits our knowledge of this for all but a few reefs– and this lack of data is even more pronounced elsewhere. Another approach would be to integrate our 4MW model with spatially explicit coral ecosystem models^2^,^59^,^60^ to explore coral response across a range of potential TC disturbance scenarios. In the meantime, predicting severe damage using the 4MW model will continue to provide a valuable basis for management decision-making following TCs and for understanding spatial variation in TC return times. The meteorological data used to drive the 4MW model is available everywhere so this robust operational model for predicting where TCs damage reefs can be used in all coral reef regions. Future work to determine levels of sea state capable of damaging other marine habitats could result in broader use of the 4MW model beyond reefs.

## Additional Information

**How to cite this article**: Puotinen, M. *et al*. A robust operational model for predicting where tropical cyclone waves damage coral reefs. *Sci. Rep.* **6**, 26009; doi: 10.1038/srep26009 (2016).

## Supplementary Material

Supplementary Information

## Figures and Tables

**Table 1:** Summary of the analysis steps for models used in this study to predict the spatial extent of tropical cyclone (TC) generated severe wave damage to coral reefs.

Model	Steps within reported method (our alterations in italics and brackets)	Thresholds
AHF	1. Identify TC intensity category and associated maximum wind speed. 2. Create damage zones for left and right sides of track to a distance based on intensity category [*switch sides due to GBR being in southern hemisphere*], assuming that each higher level category contains damage zone distance for the weaker categories (e.g. a category 5 TC will include a zone for category 5, 4, 3, 2, and 1 that are combined into an overall damage zone). 3. Estimate likely coral loss within these zones assuming linear relationship between maximum wind speed and wave heights, and based on idealised communities [*this step would further constrain predicted damage zone, omitted here as we aim to quantify extent of severe damage rather than coral loss and these are only somewhat related*]	Category 1: left- 23.6 km, right - 47.2 km; Category 2: left - 16.4 km, right - 32.8 km; Category 3: left - 10 km, right - 20 km; Category 4: left - 12.6 km, right - 25.2 km; Category 5: left - 17.4 km, 34.8 km.
FAB	1. Reconstruct hourly wind speeds using parametric TC wind model. 2. Use thresholds of the duration and maximum wind speeds known to cause severe damage to reefs during TC Ingrid (2005, n = 490) to map a damage zone.	1. Inner continental shelf reefs - maximum wind speeds > 33 m/s and at least 12 hours of gale + winds. 2. Outer shelf reefs - maximum wind speeds > 40 m/s.
4MW	1. Reconstruct hourly wind speeds using parametric TC wind model. 2. Calculate the duration of wind speeds of various strengths. 3. Calculate fetch at each site of interest. 4. Combine wind intensity, duration and fetch to determine whether a sea state capable of damaging most reefs was possible.	Significant wave height = 4 m; a ‘very rough’ sea state shown to move large reef blocks^34^.

Each model generates a spatial zone beyond which severe damage is not expected to occur, and within which severe damage is expected to be patchy. Model designations match those in Figs 1,2 and 5. A detailed description of 4MW can be found in the Supplementary material. References for models are: AHF^19^ and FAB^23^.

**Table 2:** Model comparison for tropical cyclones (TC) that tracked near the Great Barrier Reef, 1985–2015.

TC type	Max intensity (wind speed m/s)	Mean size (km)	Gale duration (hrs)	Year	Name	Reef area (%)		
						4MW	AHF	FAB
Weak – Small – Short*	29	137	8	1985	Pierre	0.0	6.7	0.0
	22	145	7	1992	Mark	0.0	0.1	0.0
	20	148	5	2004	Fritz	0.0	0.0	0.0
	18	130	5	2006	Jim	0.0	0.2	0.0
	22	136	7	2010	Tasha	0.0	1.8	0.0
	31	94	6	2013	Zane	0.0	0.0	0.0
	20	134	2	2014	Edna	0.0	0.7	0.0
Weak – Small - Typical	30	137	24	1985	Tanya	5.5	0.1	0.0
	30	65	12	1996	Ethel	0.5	6.4	0.0
	23	121	35	1998	Nathan	12.2	3.8	0.0
	29	105	12	2007	Guba	0.0	0.0	0.0
	23	120	19	2009	Ellie	4.1	2.6	0.0
	32	136	19	2010	Olga	2.5	2.2	0.0
Weak – Small - Typical	17	165	4	1989	Meena	0.0	0.0	0.0
	18	287	4	1991	Kelvin	0.0	0.0	0.0
	21	167	6	1997	Ita	0.0	2.4	0.0
	23	228	5	2001	Abigail	0.0	2.4	0.0
	24	152	8	2006	Kate	0.0	0.0	0.0
	29	176	10	2011	Anthony	3.4	4.7	0.0
Weak – Typical - Typical	19	165	14	1986	Manu	0.0	0.0	0.0
	28	187	12	2000	Tessi	0.2	3.0	0.0
Weak – Typical - Long	20	296	41	1993	Nina	1.9	0.0	0.0
Weak – Big - Typical	26	337	17	2013	Tim	0.0	0.0	0.0
Weak – Big – Long								
	32	313	71	1992	Fran	12.0	5.7	0.0
	22	439	50	1997	Justin	21.1	0.2	0.0
	18	376	54	2013	Oswald	0.0	0.5	0.0
	28	495	36	2014	Dylan	25.5	7.5	0.0
Strong – Small - Short	33	104	11	2000	Steve	2.5	3.3	0.0
	68	159	10	2006	Larry	7.0	8.5	0.9
	43	148	10	2010	Ului	5.2	9.4	0.0
Strong – Small - Long	35	137	43	1988	Charlie	8.0	3.2	0.0
Strong – Small - Typical	54	120	22	2005	Ingrid	8.5	11.9	2.6
Strong – Typical – Typical								
	52	269	25	1989	Aivu	15.5	6.8	4.9
	41	262	29	1990	Ivor	12.3	6.9	2.9
	40	154	16	1996	Celeste	9.0	9.4	4.6
	39	288	14	1999	Rona	9.5	6.0	2.3
	42	226	23	2006	Monica	19.7	4.9	3.3
	62	217	31	2009	Hamish	37.7	24.1	29.2
	58	154	19	2015	Marcia	18.7	12.4	10.8
Strong – Typical - Long	45	204	47	1986	Winifred	11.7	5.7	2.8
	52	206	85	1990	Joy	27.3	2.7	0.2
	41	199	44	1994	Rewa	20.7	12.4	1.6
	59	208	37	2014	Ita	5.6	5.6	4.2
	46	139	87	2015	Nathan	16.7	5.1	4.4
Strong – Big - Typical	61	322	17	2011	Yasi	16.6	6.9	6.5
Strong – Big – Long**	46	319	39	1993	Oliver	0.5	0.0	0.0

Each TC is classified based on the combination of its maximum intensity, mean circulation size and duration as per Fig. 3. TCs are ordered from those least likely (weak, small, short*) to most likely to cause widespread severe damage (strong, big, long**).
