# Supplementary material for: A robust operational model for predicting where tropical cyclone waves damage coral reefs
Source: Sci Rep. 2016 May 17;6:26009. doi: 10.1038/srep26009 (PMC4868967; doi:10.1038/srep26009)
Supplement: Supplementary Information [file srep26009-s1.pdf]

Supplementary Information for :

A robust operational model for predicting where tropical cyclone waves damage coral reefs

Marji Puotinen[1], Jeffrey A. Maynard[2, 3], Roger Beeden[4], Ben Radford[1], Gareth J. Williams[5]

1 - *Australian Institute of Marine Science, 35 Stirling Highway, Crawley, Western Australia 6009,*

2 – *SymbioSeas and the Marine Applied Research Center, Wilmington NC 28411, United States of America*

3 - *Laboratories d'Excellence <<CORAIL>> USR 3278 CNRS – EPHE, CRIOBE, Papetoai, Moorea, Polyne'sie Francaise*

4 - *Great Barrier Reef Marine Park Authority, Townsville, Australia,*

5 - *School of Ocean Sciences, Bangor University, Menai Bridge, Anglesey LL59 5AB, UK*

**Communicating author:*
E: m.puotinen@aims.gov.au
P: +61402345094

Contents:

1. **Expanded methods: 4MW model**
2. **Expanded results: model performance comparisons for various storm types**
3. **Literature Cited**

1. Description of the 4MW model

Coral communities are damaged by cyclone-generated waves not winds. Though wind speeds play a key role in determining wave heights, the duration of winds of a given speed as well as the extent of open water over which they can blow consistently (fetch) are equally important [1]. The 4MW model attempts to capture the essence of these relationships while remaining reasonably simple and fast to calculate. Consequently, the model is practical for researchers and managers to use in near-real time after a cyclone occurs and with meteorological data that is freely available worldwide (International Best Track Archive for Climate Stewardship, IBTrACS; https://www.ncdc.noaa.gov/ibtracs/).

Specifically, 4MW predicts whether a sea state sufficient to severely damage most vulnerable coral communities was possible during each TC at each pixel across the study area. While long period swell can be generated by some cyclones under certain conditions, an extensive dataset of 440 cyclones examined with satellite altimeter data recently demonstrated that most waves move forward with the TC [2]. Thus, the sea state built by locally generated cyclone winds is our focus. Further, recent studies have confirmed that the GBR acts as almost a complete barrier to long period swell [3] – this means that only the exposed slopes of the outermost shelf reefs are likely exposed to whatever long period swell is occasionally generated by cyclones near but seaward of the GBR.

A sea state, at any given time, is comprised of waves of varying heights [1]. Significant wave height (Hs) – the mean wave height of the one-third highest waves - is an indicator of sea state [1]. The sea state required to damage coral communities varies in space and time with factors like species composition, community structure and habitat geomorphology. Given this, we modelled a level of sea state severity at which severe damage to *most* coral reef

colonies becomes likely ('very rough' sea state). The roughness of a given sea state is characterised based on significant wave height (H_S) using the internationally recognised Douglas Sea Scale, ranging from calm (degree 0, no waves) to phenomenal (degree 9, 14+ m waves). On this scale, sea conditions are termed 'very rough' when H_S = 4m. This means that one-third of waves will be at least 4 m high, with maximum waves up to ~10 m. This 'very rough' sea state is at least one-third more energetic than calm conditions and has been shown to move entire reef blocks onto the reef flat [4].

The duration of wind speeds from 15 m s^{-1} to 33 m s^{-1} in 1 m s^{-1} increments required to generate very rough (H_S = 4) seas and the required fetch for each was obtained using empirical relationships published by the US Army Corps of Engineers (USACE Shore Protection Manual, volume 3, Fig. 7.8) and is shown in Table S1.

Table S1: Conditions required to generate 'very rough' seas characterised by significant wave heights (Hs) equal to 4 metres. These are based on relationships between wind speed, wind duration and fetch established by the US Army Corps of Engineers and reported in their Shore Protection Manual. Winds must persist increasingly longer and blow unobstructed over increasingly greater distances (fetch) to generate very rough seas as wind speed declines.

**Wind Speed** (m s^{-1})	**Duration (hrs)**	**Fetch (km)**
15	16	275
16	14	250
17	12.25	220
18	10	175
19	8.25	145
20	7.25	120
21	6	95
22	5	77
23	4.6	71
24	4	66
25	3.7	59
26	3.3	57
27	3	49

28	2.7	43
29	2.4	38
30	2.25	36
31	2	33
32	1.9	29
33	1.75	28

For each cyclone, we used a parametric wind model [5] anchored in the outer radii of gale-force winds to calculate 10-minute maximum wind speeds at every 4-km pixel across the GBR for every hour along the track (as per [6], [7]). An asymmetry correction [8] was applied and the resulting wind speeds were scaled to fit within the gale radii. This produced a map of wind speed every hour for each TC. From these maps, the duration (hrs) of winds of each speed every 1 m $s^{-1}$from 15 m s^{-1} to 33 m $s^{-1}$was calculated. The lower bound (15 m s^{-1}) was chosen because it is the lowest wind speed capable of generating Hs = 4m given sufficient fetch and duration. The upper bound (33 m s^{-1}) was chosen because it is the highest wind speed for which a specific wind duration or fetch is needed to generate Hs = 4m. The wind speed (1 map per TC) and persistence (18 maps per TC) maps were then used to characterise whether a 'very rough' sea state was possible during each TC at each pixel. For each of the surveyed sites from each of the 7 test cyclones, we then calculated the unobstructed sea distance in each of 48 directions around the site (spaced at 7.5 degree intervals) using GREMO software [9]. We used this data with time series of wind speed and direction during each cyclone at each surveyed site to determine the fetch up to 22.5 degrees on either side of the dominant incoming wind direction during peak conditions of the storm. We then manually adjusted the Hs estimates for those locations where fetch was insufficient during the cyclone to generate the 4 m sea state.

The wind modelling was based on a spatial database of TCs near the GBR from 1985-2015 [10] interpolated to a one-hourly interval. The direction of forward motion and translation speed were calculated as per [11]. Where necessary, standard values for radius to gales were taken from [12] and regionally adjusted as per [13].

2. Expanded results – model performance comparisons for various TC types.

Table S2. Summary of model performance for the 7 TCs for which damage field data were available (see also Fig. 2). Top performers are the model(s) with the highest true positive rates that also meet or exceed the pAUC model performance benchmark.

Cyclone	Cyclone type	Top performer	Model performance benchmarks	
			True positive rate >= 0.9	*pAUC >= 0.7*
Justin	Weak, large, long-lived	4MW	4MW	4MW, AHF
Yasi	Strong, large, typical duration	4MW	4MW	4MW
Joy	Strong, typical size, long-lived	4MW	4MW	4MW, AHF
Ita	Strong, typical size, long lived	FAB	4MW, AHF, FAB	AHF, FAB
Larry	Strong, small, short-lived	Tie: ALL	4MW, AHF, FAB	4MW, AHF, FAB
Ingrid	Strong, small, typical duration	4MW	4MW, AHF	4MW
Ivor	Strong, typical size, typical duration	Tie: 4MW - AHF	4MW, AHF	4MW, AHF, FAB

Weak, large, and long-lived: Justin was the second-largest recorded TC in the GBR over the study period (Fig. 4) while it was located well out in the Coral Sea (Fig. 2-c). Justin

generated gale+ winds that persisted for weeks across two-thirds of the GBR even though it was weak (Fig. 4, Table S2) and located 100s of km away from the nearest reefs. Consequently, 4MW (true positive rate = 1) outperformed AHF and FAB, both of which failed to capture any of the observed severe damage (true positive rates, 0.2 and 0). Similarly, only 4MW and AFH met the 0.7 pAUC benchmark (4MW- 1, AHF – 0.84). Weak, large and long-lived TCs revisit the GBR every 8.3 years (Fig. 4).

Strong, large, and typical duration: 4MW clearly outperformed AHF for large and strong Yasi (Fig. 2-f, true positive rate = 1 versus = 0.7), and only 4MW met the pAUC benchmark of 0.7 (0.71). This difference in true positive rates was less dramatic than that of large but weak and long lasting TC Justin because Yasi tracked near reefs while strong enough to trigger AHF to define a damage zone. Further, Yasi generated extreme conditions at reefs for less than half as long as Justin (Fig. 4, 17 hours versus 50 hours – Table S2) – limiting the consequences of AHF not considering the impact of long-lasting lower level winds on sea state. Nonetheless, the 4MW damage zone for Yasi was still considerably larger than that of AHF (Fig. 2-f). Strong and big TCs that are typical in duration like Yasi are less common, predicted to return to the GBR every 16 years (Fig. 4, Table S2).

Strong, typical, and long-lived: Though typical in size, TC Joy's gale+ winds lasted the second longest of any TC in the GBR from 1985-2015 (Fig. 4, 85 hours - Table S2). This enabled the development of rougher seas than would be expected given the peak wind speeds, and is the key reason why 4MW's true positive rate for Joy (1) outperformed the other models, particularly AHF (0.5). 4MW and AHF met the pAUC benchmark (4MW – 0.9, AHF – 0.79).

For Ita (Fig. 2-g), which was also strong and somewhat long-lived (gale winds in GBR of 37 hours - Table S2), 4MW's true positive rate (0.89) was slightly higher than that of AHF (0.87), but both were lower than that of FAB (0.99 - Fig. 2-g). This occurred because a cluster of surveyed sites at the Low Isles were severely damaged despite being highly sheltered in almost all directions which caused them to drop out of the 4MW damage zone but be retained in the FAB damage zone which does not attempt to consider fetch. If not for this cluster of sites, 4MW's true positive rate would have been 1. This highlights the failure of damage zone models to consider local scale factors like variation in coral community fragility – the normally sheltered Low Isles sites would require less wave energy to be damaged (and thus less fetch to build the lower sea state needed for damage). FAB (0.99) performed better for Ita than for other TCs because Ita was very intense while very near the surveyed sites. In this case, 4MW, AHF and FAB all met the 0.9 benchmark for true positive rate, and AHF and FAB met the 0.7 benchmark for pAUC.

In general, AHF appears to under-predict the extent of potential damage from strong and long-lived TCs (Table S2) - the 4MW damage zones are much more extensive than those of AHF (Fig. 2-b, f, g). Strong and long-lived TCs revisit the GBR once every 6.7 years (Fig. 4).

Strong, small, and short-lived. Severe damage from Larry (Fig. 2-e) was extremely localised despite its high intensity due to a very fast translation speed. Some reefs experienced cyclonic conditions for as little as 20 minutes. Thus, it seems possible that the damage zones predicted by all three models (all with true positive rates of 1)

overestimated the spatial extent of actual damage for this unusually short-lived TC (a maximum of only 10 hours of gales at reefs, Fig. 4, Table S2), though 4MW and FAB both met the 0.7 benchmark for pAUC. TCs like this - that are strong, small and short-lived - track near or within the GBR about once per decade (Fig. 4, Table S2).

Strong, small, and typical duration: In contrast, damage field data from small cyclone Ingrid (Fig. 2-d) matched well to the damage zones predicted by both 4MW and AHF, both of which had true positive rates of 1. However, only 4MW met the 0.7 benchmark for pAUC. Though not as intense as Larry (Fig. 4), Ingrid generated gales in the GBR for nearly twice as long (22 versus 12 hours – Table S2). TCs like this are rare in the GBR – predicted to occur every 31.5 years.

Strong, typical size, and typical duration: AHF equalled 4MW's performance for typically-sized Ivor (Fig. 2-a) because AHF's failure to consider persistent lower level winds far from the track was less relevant for this type of TC (Table S2). FAB's true positive rate for Ivor (0.1) was well below the benchmark because Ivor was not strong enough to trigger a large enough damage zone to correctly predict enough observed damage. All three models met the pAUC benchmark. These TCs are predicted to be common - revisiting the GBR every 4.9 years.

Weak, typical and short-lived and Strong, typical and long-lived. This section differs from those presented above. No field data on damage were available for the GBR for these types of storms. Here, we describe differences among models in the extents of the predicted damage zones. AHF predicted the existence of a TC damage zone when 4MW and FAB did not for 71% of TCs that were weak, small and short (5 of 7 cases,

Table S2). A similar result occurred for TCs that were weak, typical in size and short (Table 2). It is plausible that AHF over-predicts damage for TCs that are weak and short-lived (Table S2) because winds are unlikely to have sufficient time to generate the sea states implied by the TC intensity that triggers the creation of the AHF damage zone. These TCs are common in the GBR – revisiting the region every 2.8 years.

3. Literature Cited

1 Denny, M. *Biology and the Mechanics of the Wave-swept environment.* (Princeton University Press, Princeton, NJ., 1988)

2 Young, I. R. & Vinoth, J. *A parametric model for tropical cyclone waves* in ASME 2013 32nd International Conference on Ocean, Offshore and Arctic Engineering (pp. V02AT02A002-V02AT02A002). (American Society of Mechanical Engineers, 2013)

3 Gallop, S. L., Young, I. R., Ranasinghe, R., Durrant, T. H. & Haigh, I. D. The large-scale influence of the Great Barrier Reef matrix on wave attenuation. *Coral Reefs*, **33**(4),1167-1178 (2014).

4 Goto, K., Okada, K. & Imamura F. Characteristics and hydrodynamics of boulders transported by storm waves at Kudaka Island, Japan. *Mar. Geol.*, **262**, 14-24 (2009).

5 Holland, G. J., Belanger, J. I. & Fritz, A. A Revised Model for Radial Profiles of Hurricane Winds. *Mon. Weather Rev.*, **138**, 4393-4401 (2010).

6 Puotinen, M. L. Modelling the risk of cyclone wave damage to coral reefs using GIS: a case study of the Great Barrier Reef, 1969-2003. *Int. J. GIS,* **21**,97-120 (2007).

7 Fabricius, K. E., De'ath, G., Puotinen, M. L., Done, T., Cooper, T. F. & Burgess, S. C. Disturbance gradients on inshore and offshore coral reefs caused by a severe tropical cyclone. *Limnol. Oceanog.*, **53**, 690-704 (2008).

8 McConochie, J. D., Hardy, T. A. & Mason, L. B. Modelling tropical cyclone over-water wind and pressure fields. *Ocean Engineering*, **31**,1757-1782 (2004) .

9 Pepper, A. & Puotinen, M. L. *GREMO: A GIS-based generic model for estimating relative wave exposure* in The 18th World IMACS Congress and MODSIM09 International Congress on Modelling and Simulation (pp. 1964-1970). (Cairns, Australia: Modelling and Simulation Society of Australia and New Zealand and IMACS 2009).

10 Knapp, K. R., Kruk, M. C., Levinson, D. H., Diamond, H. J. & Neumann, C. J. The International Best Track Archive for Climate Stewardship (IBTrACS): Unifying tropical cyclone best track data. *Bull. Amer. Met. Soc.*, **91**,363–376 (2010).

11 Mei, W., Pasquero, C. & Primeau, F. The effect of translation speed upon the intensity of tropical cyclones over the tropical ocean. *Geophys. Res. Lett.*, **39**, L07801 (2012).

12 Moyer A. C., Evans, J. L. & Powell, M. Comparison of observed gale radius statistics. *Meteorol. Atmos. Phys.*, **97**, 41–55 (2007).

13 Chavas, D. R. & Emanuel, K. A. A QuikSCAT climatology of tropical cyclone size. *Geophys. Res. Lett.*, **37**, L18816, doi:10.1029/2010GL044558 (2010).
